# Effects of mild and moderate renal dysfunction on pharmacokinetics, pharmacodynamics, and safety of dotinurad: a novel selective urate reabsorption inhibitor

**DOI:** 10.1007/s10157-019-01825-3

**Published:** 2019-12-10

**Authors:** Hiroyuki Fukase, Daisuke Okui, Tomomitsu Sasaki, Masahiko Fushimi, Tetsuo Ohashi, Tatsuo Hosoya

**Affiliations:** 1 Clinical Research Hospital Tokyo, NT-Building Level 3, 3-87-4, Hara-machi, Shinjuku-ku, Tokyo, 162-0053 Japan; 2 Medical R&D Division, Development Department, Fuji Yakuhin Co., Ltd., 4-383, Sakuragi-cho, Omiya-ku, Saitama-shi, Saitama, 330-9508 Japan; 3 Jikei University School of Medicine, 3-25-8, Nishi-Shimbashi, Minato-ku, Tokyo, 105-8461 Japan

**Keywords:** Dotinurad, Renal dysfunction, URAT1 inhibitor, Selective urate reabsorption inhibitor, Pharmacokinetics, Pharmacodynamics

## Abstract

**Background:**

Dotinurad, a novel selective urate reabsorption inhibitor, exerts a serum uric acid-lowering effect by selectively inhibiting urate transporter 1 (URAT1) in patients with hyperuricemia. It is generally known that the progression of renal dysfunction is associated with a reduction in the serum uric acid-lowering effects of uricosuric drugs. We, therefore, investigated the pharmacokinetics (PK), pharmacodynamics (PD), and safety of dotinurad in subjects with renal dysfunction.

**Methods:**

This was a parallel-group, open-label, single-dose clinical pharmacology study. Dotinurad (1 mg) was administered once, orally to subjects with mild (estimated glomerular filtration rate [eGFR], ≥ 60 to < 90 mL/min/1.73 m^2^) or moderate (eGFR, ≥ 30 to < 60 mL/min/1.73 m^2^) renal dysfunction or normal (eGFR, ≥ 90 mL/min/1.73 m^2^) renal function.

**Results:**

The time-course of mean plasma concentration of dotinurad had similar profiles across the groups. Regarding PK, there was no significant difference between the renal dysfunction groups and normal renal function group. Regarding PD, the maximum reduction rate in serum uric acid levels and the fractional uric acid excretion (FE) ratio (FE_0–24_/FE_−24–0_) were significantly lower in the moderate renal dysfunction group than in the normal renal function group. However, other PD parameters were not significantly different among the groups. No notable adverse events or adverse drug reactions were observed in this study.

**Conclusion:**

These results suggested that no dose adjustment might be necessary when administering dotinurad to patients with mild-to-moderate renal dysfunction.

ClinicalTrials.gov Identifier: NCT02347046.

**Electronic supplementary material:**

The online version of this article (10.1007/s10157-019-01825-3) contains supplementary material, which is available to authorized users.

## Introduction

Hyperuricemia, defined in Japan as a serum uric acid level > 7.0 mg/dL [1, 2], is known to cause diseases related to uric acid deposition, such as gouty arthritis and renal disorders. In addition, hyperuricemia has been reported to be associated with the onset and progression of chronic kidney disease [3] and identified as a risk factor for kidney failure [4]. Correcting and appropriately managing serum uric acid levels have been considered important for preventing these diseases and it is thought that treatment of hyperuricemia will become increasingly important in the future.

Lifestyle modification is considered vital for treating hyperuricemia. However, because patients with recurrent gouty arthritis or gouty tophi find it difficult to alleviate their symptoms by lifestyle modification alone, they are advised to maintain a serum uric acid level of less than 6.0 mg/dL using antihyperuricemic agents. Hyperuricemia is mainly classified into three types: excess uric acid production “overproduction type”; reduced uric acid excretion “underexcretion type”; and a mixture of both “combined type”. The basic principle of hyperuricemia treatment in the Japanese guidelines for the management of hyperuricemia and gout is the use of xanthine oxidase inhibitors (XOIs) (i.e., allopurinol, febuxostat) for “overproduction type” and uricosuric drugs (i.e., benzbromarone, probenecid, and bucolome) for “underexcretion type” [5]. However, XOIs have been recommended for patients with renal dysfunction, regardless of the type of hyperuricemia [6]. The progression of renal dysfunction results in markedly reduced uric acid-lowering effects of probenecid, bucolome [7], and lesinurad [8]. In contrast, even in patients with progressive renal dysfunction, the uric acid-lowering effect of benzbromarone is not attenuated and serum uric acid levels can be lowered by increasing the dose [7]. However, the safety of benzbromarone in patients with moderate renal dysfunction has not been fully confirmed [9]. Therefore, according to the Japanese management guidelines, uricosuric drugs are not recommended for patients with renal dysfunction because of the decline in effectiveness and concerns about safety.

In recent cohort study, it has been reported that hyperuricemia is associated with the progression of renal dysfunction [10]; many hyperuricemic patients are thus estimated to have renal dysfunction. In addition, it is generally known that renal function declines with age. According to the Japanese management guidelines, most outpatients with gout are 50 years or older [7]. Therefore, the use of treatment for hyperuricemia is expected to increase with age.

In this context, it was considered necessary to investigate the pharmacokinetics (PK), pharmacodynamics (PD), and safety of dotinurad in subjects with renal dysfunction.

## Methods

### Study design

This parallel-group, open-label, single-dose clinical pharmacology study was conducted to examine the PK, PD, and safety of dotinurad in subjects with renal dysfunction. After informed consent was obtained, a screening test was conducted to confirm subject eligibility within 30 days prior to dotinurad administration. During the screening, confirmation of medical history, physical examination, clinical laboratory tests, electrocardiogram (ECG), abdominal ultrasonography, and abdominal X-ray were performed and the estimated glomerular filtration rate (eGFR) was calculated. The eGFR of subjects judged to be eligible at the time of screening was calculated again on Day−1 (the day before dotinurad administration) and the subjects were assigned to one of three groups (mild renal dysfunction group, moderate renal dysfunction group, and normal renal function group), according to the eGFR. Mild renal dysfunction was defined by an eGFR of ≥ 60 to < 90 mL/min/1.73 m^2^. Moderate renal dysfunction was defined as an eGFR of ≥ 30 to < 60 mL/min/1.73 m^2^. Normal renal function was defined as an eGFR of ≥ 90 mL/min/1.73 m^2^. The subjects were admitted to the institution on Day−1, where they remained until completion of the study, 48 h after the administration of dotinurad (Day 3). After subjects fasted for at least 10 h, dotinurad (1 mg) was administered once, orally on the morning of Day 1 with 180 mL of water. Follow-up testing was performed 5–10 days after the administration of dotinurad.

### Inclusion and exclusion criteria

The inclusion criteria for this study were as follows: male Japanese subjects aged 20 years or older at the time of informed consent; assignment of subjects to the same group—one of normal renal function, mild renal dysfunction or moderate renal dysfunction—at the time of the screening test and on Day−1; variation in serum creatinine of no more than 0.2 mg/dL; absence of contraindications to inclusion in this study as a result of examination of the following items, performed within 30 days before administration of the investigational drugs: (1) findings on medical examination, (2) physical examination, body mass index (BMI) and assessment of vital signs, (3) ECG, (4) abdominal ultrasonography, plain abdominal x-ray, and (5) laboratory tests; subjects with a BMI of not less than 18.5 and less than 30.0.

Exclusion criteria were as follows: disorders of the digestive tract, heart, or liver; severe renal dysfunction; history of surgery involving the digestive organs (digestive tract, liver, gallbladder, bile duct, and pancreas) or kidneys, excluding surgery not considered to affect drug absorption (such as appendectomy and hemorrhoidectomy); renal calculus or clinical manifestations such as hematuria or back pain that might be due to a urinary calculus as assessed by abdominal ultrasonography or plain abdominal X-ray in the screening period; consumption of St. John’s wort or foods containing St. John’s wort within 4 weeks of administration of the dotinurad; consumption of grapefruit or foods containing grapefruit within 7 days of dotinurad administration; use of other drugs (including over-the-counter drugs, vitamin preparations, and energy drinks) within 7 days of dotinurad administration; alcohol consumption within 3 days of dotinurad administration; use of investigational drugs other than dotinurad within 12 weeks of dotinurad administration (or 4 weeks in the case of patch tests);previous participation in a study of dotinurad with administration of the investigational drug.

### Blood and urine sample collection

Blood samples for PK and PD analyses were collected 12 times, namely before administration and 0.5, 1, 2, 3, 4, 6, 8, 12, 24, 36, and 48 h after administration. Urine samples for PK and PD analyses were collected three times at 24-h intervals from 24 h before administration to 48 h after administration.

### Analytical methods

The dotinurad and its metabolites’ concentration in plasma and urine were measured using liquid chromatography–tandem mass spectrometry (LC–MS/MS) at the research institute, Fuji Yakuhin Co., Ltd.

The Agilent 1100 series (Agilent Technologies, USA) was used for the liquid chromatography; API3000 (AB SCIEX, USA) was used for the mass spectrometry; and Inertsil ODS-3 (150 mm × 2.1 mm, 3 μm, GL Sciences Inc., Japan) was used for the analysis column. Measurements were made using 5 mmol/L ammonium acetate solution (pH 4.0)/methanol (50:50) in the mobile phase. The lower limit of quantification for dotinurad and its metabolites in human plasma and urine was 1 ng/mL.

### Pharmacokinetic analyses

The PK samples were analyzed at Fuji Yakuhin Co., Ltd (Saitama, Japan).

The PK parameters of dotinurad and its metabolites were estimated using a non-compartmental method. Plasma PK parameters included time to reach maximum plasma concentration (*T*_max_), maximum observed plasma concentration (*C*_max_), area under the plasma concentration-time curve (AUC), AUC from zero to infinity (AUC_0–inf_), apparent total body clearance (CL/F), terminal elimination half-life (*T*_1/2_), apparent volume of distribution (*V*_d_/F), elimination rate constant (kel), and mean residence time from time zero to the last measurable sample point (MRT_0–*t*_). Urinary PK parameters included the amount of unchanged drug excreted in urine over each collection interval (Ae) and fraction of dose excreted in urine expressed as a percentage (fe). These PK parameters were derived using WinNonlin Professional, version 6.2 (Pharsight Corporation, Mountain View, CA, USA).

### Pharmacodynamic analyses

Serum and urinary uric acid concentrations were measured at the clinical trial institution and PD parameters were calculated from the measurement results. Serum PD parameters included the maximal change in serum uric acid levels from baseline (∆EC_max_) and mean time-matched change in serum uric acid levels from baseline (∆AUEC). Urinary PD parameters included the amount of uric acid in urine (Ae_0–t, ua_); renal clearance of uric acid (CL_R_); creatinine clearance (Ccr), which was calculated as Ae divided by plasma uric acid AUC over the same time interval, and fractional excretion of uric acid (FE), which was calculated as (CL_R_/Ccr) × 100.

### Safety evaluations

Clinical investigators conducted safety assessments including vital signs, ECG, clinical laboratory tests, and clinical examinations throughout the study period and recorded adverse events (AEs). All AEs were classified according to the system organ class and preferred term (MedDRA/J version 17.1; Japanese Maintenance Organization, Tokyo, Japan) and evaluated in terms of their potential causality with the study drug, severity, and seriousness. AEs judged to have a causal relationship with the study drug were defined as adverse drug reactions (ADRs).

### Statistical analyses

All statistical analyses were performed using SAS software (version 9.2.; SAS Institute Inc., Cary, NC, USA). All subjects who received dotinurad and who had evaluable PK and PD data, excluding those who did not meet the inclusion and exclusion criteria, were included in PK and PD analysis sets. Safety analysis sets consisted of all subjects who received dotinurad. Descriptive statistics for PD and safety population data included number of subjects, arithmetic mean, minimum, maximum, and standard deviation (SD). Statistical tests for baseline characteristics were two-sided and *P* < 0.15 denoted statistical significance. The other statistical tests and confidence intervals were two-sided and *P* < 0.05 denoted statistical significance.

Summary statistics are provided for PK and PD parameters in each group. Regarding the PK and PD parameters of dotinurad, to evaluate the effect of renal dysfunction, Dunnett’s test was used to compare the mild and moderate renal dysfunction groups with the normal renal function group. Differences of means with their corresponding 90% confidence intervals and estimates of geometric mean ratios via anti-logarithmic transformation with their corresponding 90% confidence intervals were generated for *C*_max_, AUC, and AUC_0–inf_.

## Results

### Subjects

In this study, 18 adult male subjects were enrolled with six subjects assigned to each of the three groups. All subjects received dotinurad and completed this study. However, because one subject in the moderate renal dysfunction group deviated from the study protocol (use of other drugs in the 7 days before administration of dotinurad), data for this subject were excluded from the PK and PD analyses. Therefore, 17 subjects were included in the PK and PD evaluation and 18 subjects were included in the safety evaluation.

Demographic and other baseline characteristics of each group are shown in Table 1. Bias in eGFR was observed because of the degree of renal function in each group, but no bias was observed for other demographic and baseline characteristics (height, weight, and BMI).

**Table 1:** Summary of demographic data

Parameters	Renal function			*P* value
	Normal (*n* = 6)	Mild dysfunction (*n* = 6)	Moderate dysfunction (*n* = 5)	
Age (year)	22.7 ± 1.6	49.3 ± 16.3	74.8 ± 3.3	–
Height (cm)	170.57 ± 6.00	167.08 ± 10.92	166.38 ± 5.34	0.645
Weight (kg)	63.37 ± 6.46	65.77 ± 11.38	61.06 ± 3.27	0.633
BMI (kg/m^2^)	21.70 ± 1.58	23.42 ± 2.20	22.06 ± 1.80	0.287
eGFR (mL/min/1.73 m^2^)	102.2 ± 6.7	66.5 ± 4.5	50.2 ± 12.0*	< 0.001*
Serum uric acid level (mg/dL)	5.92 ± 0.44	6.20 ± 1.74	7.36 ± 2.24	–

*BMI* body mass index

*P < 0.15 (analysis of variance)

Data presented as mean ± SD

### Pharmacokinetics

#### Dotinurad (unchanged)

**Fig. 1:**
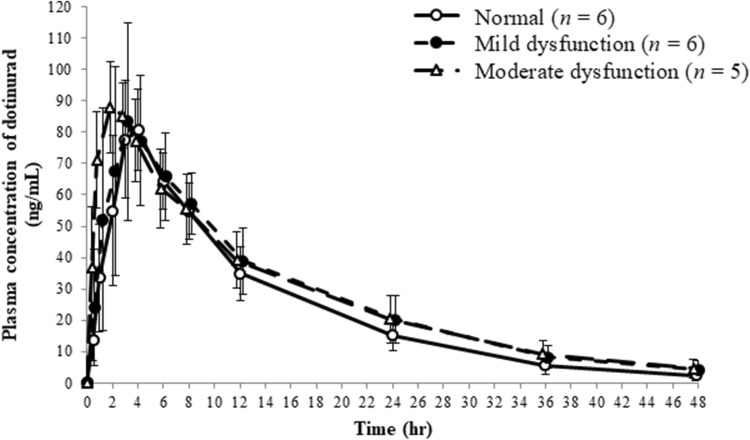
The time-course of concentration of dotinurad. Error bars indicate standard deviation

The time-course of mean plasma concentration of dotinurad in each group is shown in Fig. 1 and the plasma and urine PK parameters of dotinurad in each group are shown in Table 2. The time-course of mean plasma concentration of dotinurad was not affected by the degree of renal dysfunction. The PK parameters (mean ± SD) of the normal renal function and mild and moderate renal dysfunction groups were as follows: *C*_max_ 85.67 ± 10.65, 88.73 ± 22.74, and 88.38 ± 14.39 ng/mL, respectively; AUC_0–inf_ 1157.32 ± 269.46, 1366.57 ± 427.94, and 1428.54 ± 379.58 ng h/mL, respectively; CL_tot_/F 0.91 ± 0.25, 0.81 ± 0.33, and 0.75 ± 0.25 L/h, respectively; fe_0–24_ 0.97 ± 0.35%, 0.88 ± 0.32%, and 0.52 ± 0.16%, respectively. No urinary excretion of dotinurad was observed after 24 h of administration in any group. The mean plasma and urine PK parameters of dotinurad were comparable among the groups and no significant differences of parameters (*C*_max_, *T*_max_, *T*_1/2_, AUC_0–inf_, CL_tot_/F) were observed between the renal dysfunction groups and normal renal function group.

**Table 2:** The plasma and urine PK parameters of dotinurad and its major metabolites

Drug and its major metabolites	Renal function			*P* value
Parameters	Normal (*n* = 6)	Mild dysfunction (*n* = 6)	Moderate dysfunction (*n* = 5)	Mild/Moderate
Dotinurad (unchanged)				
*C*_max_ (ng/mL)	85.67 ± 10.65	88.73 ± 22.74	88.38 ± 14.39	0.931/0.951
*T*_max_ (h)	3.50 ± 0.55	3.00 ± 1.67	2.60 ± 0.55	0.655/0.324
*T*_1/2_ (h)	8.75 ± 1.80	10.29 ± 1.50	10.95 ± 2.17	0.274/0.116
AUC_0-inf_ (ng h/mL)	1157.32 ± 269.46	1366.57 ± 427.94	1428.54 ± 379.58	0.524/0.389
Cl_tot_/F(L/h)	0.911 ± 0.251	0.813 ± 0.331	0.752 ± 0.251	0.780/0.567
kel (1/h)	0.0822 ± 0.0174	0.0687 ± 0.0111	0.0660 ± 0.0168	–
*V*_d_/F (L)	11.05 ± 1.44	11.51 ± 2.56	11.27 ± 1.23	–
MRT_0–*t*_ (h)	12.05 ± 1.31	13.03 ± 1.33	12.90 ± 2.01	–
Ae_0–24_ (μg)	9.66 ± 3.47	8.80 ± 3.23	5.23 ± 1.63	–
Fe_0–24_ (%)	0.97 ± 0.35	0.88 ± 0.32	0.52 ± 0.16	–
Glucuronate conjugate				
*C*_max_ (ng/mL)	NC	1.23 ± 0.15	1.75 ± 0.48	–
*T*_max_ (h)	NC	2.00 ± 1.15	2.50 ± 0.58	–
MRT_0–*t*_ (h)	NC	3.46 ± 1.26	5.08 ± 3.70	–
Ae_0–24_ (μg)	414.69 ± 19.33	377.30 ± 60.38	284.31 ± 56.86	–
Fe_0–24_ (%)	27.80 ± 1.30	25.29 ± 4.05	19.06 ± 3.81	–
Sulfate conjugate				
*C*_max_ (ng/mL)	2.48 ± 0.80	2.57 ± 0.71	3.16 ± 1.20	–
*T*_max_ (h)	3.67 ± 0.52	3.67 ± 2.73	4.60 ± 2.41	–
MRT_0–*t*_ (h)	5.00 ± 1.18	4.33 ± 1.40	5.29 ± 0.88	–
Ae_0–24_ (μg)	237.92 ± 71.53	156.35 ± 71.48	141.08 ± 86.32	–
fe_0–24_ (%)	19.45 ± 5.85	12.78 ± 5.84	11.53 ± 7.06	–

*NC* non calculate

Data presented as mean ± SD

Ae_0–24_, amount of dotinurad and its metabolites excreted in urine from 0 to 24 h after administration

### Major metabolites of dotinurad

The plasma and urine PK parameters of dotinurad and its major metabolites (glucuronate and sulfate conjugates) in each group are shown in Table 2. The mean *C*_max_ of glucuronate and sulfate conjugates in each group were less than 2.0 and 4.0 ng/mL, respectively. The AUC_0–inf_ of both glucuronate and sulfate conjugates in each group were not calculated. In contrast, the urinary excretion rates (0–24 h) of glucuronate (mean ± SD) for the normal renal function and mild and moderate renal dysfunction groups were 27.80 ± 1.30%, 25.29 ± 4.05%, and 19.06 ± 3.81%, respectively, and those of sulfate-conjugates were 19.45 ± 5.85%, 12.78 ± 5.84%, and 11.53 ± 7.06%, respectively.

### Pharmacodynamics

#### Serum uric acid

**Fig. 2:**
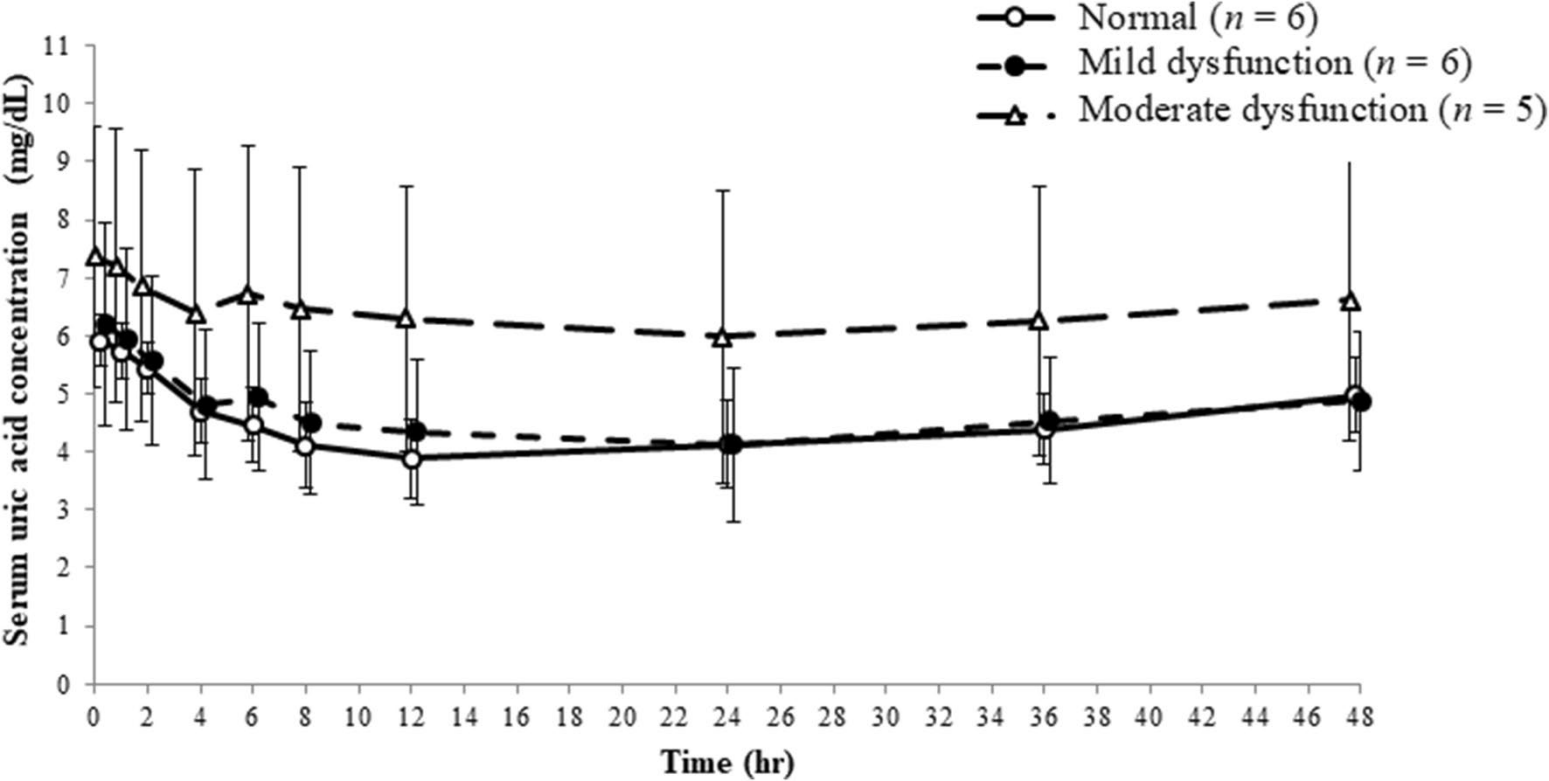
The time-course of serum uric acid concentrations. Error bars indicate standard deviation

The time-course of mean serum uric acid concentrations and summary statistics in each group are shown in Fig. 2 and Table 3, respectively. The serum PD parameters (ΔEC_max_, ΔAUEC_0–48_, and maximum reduction rate) in the normal renal function and mild and moderate renal dysfunction groups were as follows: ΔEC_max_ – 2.07 ± 0.34, − 2.07 ± 0.53, and − 1.42 ± 0.43 mg/dL, respectively; ΔAUEC_0–48_ − 73.41 ± 12.99, − 79.12 ± 27.23, and − 49.49 ± 14.59 mg·hr/dL; maximum reduction rate 35.25 ± 7.24%, 33.94 ± 6.05%, and 21.31 ± 10.40%, respectively. The maximum reduction rate was significantly different between the normal renal function and moderate renal dysfunction groups, but no significant differences were observed for other PD parameters in the renal dysfunction groups, in comparison with those in the normal group.

**Table 3:** Plasma and urine pharmacodynamic parameters

Parameters	Renal function			*P* value
	Normal (*n* = 6)	Mild dysfunction (*n* = 6)	Moderate dysfunction (*n* = 5)	Mild/moderate
ΔEC_max_ (mg/dL)	− 2.07 ± 0.34	− 2.07 ± 0.53	− 1.42 ± 0.43	1.000/0.055
ΔAUEC_0–48_ (mg h/dL)	− 73.41 ± 12.99	− 79.12 ± 27.23	− 49.49 ± 14.59	0.837/0.113
Maximum reduction rate (%)	35.25 ± 7.24	33.94 ± 6.05	21.31 ± 10.40	0.943/0.021*
Ae_0–24, ua_ (mg)	980.67 ± 131.71	982.83 ± 100.22	811.75 ± 81.23	–
CL_R0–24_ (mL/min)	16.05 ± 2.92	15.80 ± 4.38	9.96 ± 3.86	–
FE_−24–0_ (%)	4.01 ± 0.72	5.77 ± 2.41	5.70 ± 1.97	0.203/0.254
FE_0-24_ (%)	13.30 ± 2.67	16.10 ± 6.44	14.17 ± 5.63	0.557/0.945
FE_0-24_/FE_−24–0_	3.34 ± 0.51	2.86 ± 0.50	2.46 ± 0.36	0.168/0.015*

*P < 0.05

Data presented as mean ± SD

Ae_0–24,ua_, amount of uric acid excreted in urine from 0 to 24 h after administration

#### Urinary uric acid

**Fig. 3:**
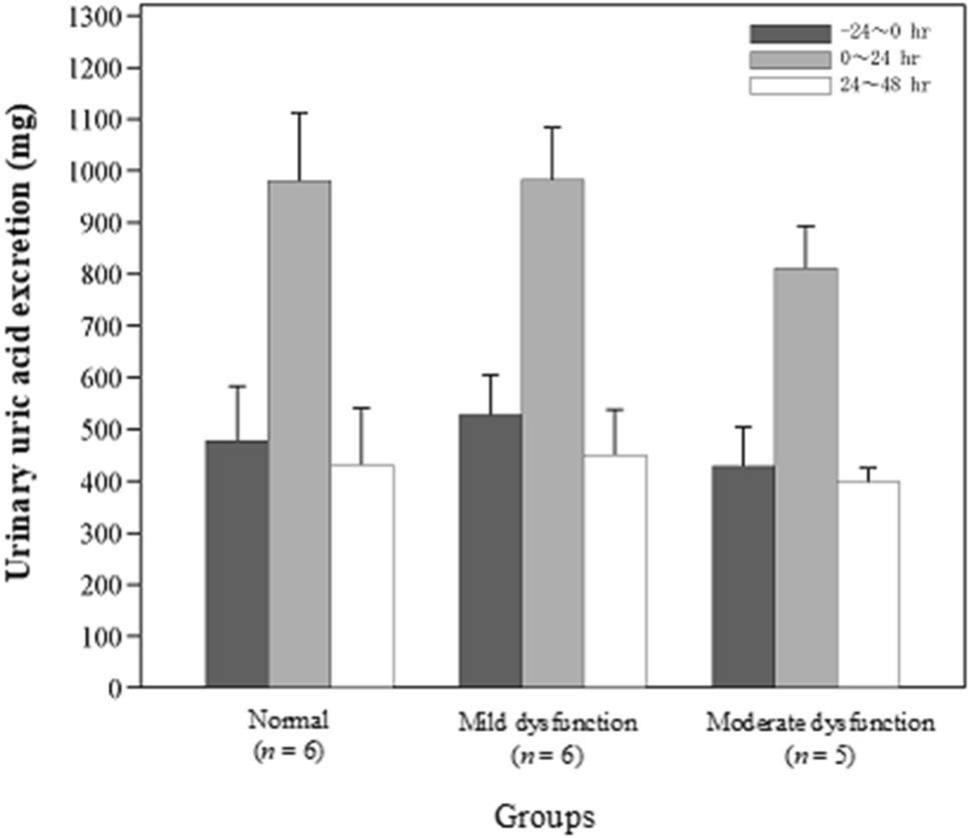
Change in urinary uric acid excretion. Error bars indicate standard deviation

The time-course of Ae_0–24, ua_ and summary statistics in each group are shown in Fig. 3 and Table 3, respectively. The urinary PD parameters (Ae_0–24, ua_, CL_R0-24_, FE_0-24_/FE_−24–0_) in the normal renal function and mild and moderate renal dysfunction groups were as follows: Ae_0-24, ua_ 980.67 ± 131.71, 982.83 ± 100.22, and 811.75 ± 81.23 mg, respectively; CL_R0–24_ 16.05 ± 2.92, 15.80 ± 4.38, and 9.96 ± 3.86 mL/min, respectively; and FE_0–24_/FE_−24-0_ 3.34 ± 0.51%, 2.86 ± 0.50%, and 2.46 ± 0.36%, respectively. FE_0–24_/FE_−24–0_ was significantly different between the normal renal function and moderate renal dysfunction groups, whereas no differences were observed for other urine PD parameters in renal dysfunction groups in comparison with those in the normal group.

### Safety

AEs were not observed in the normal renal function and mild renal dysfunction groups; however, six AEs were observed in two subjects in the moderate renal dysfunction group (Table 4). No serious AEs were recorded. Among the AEs, gouty arthritis (one event in one subject) was judged by the investigator to be an ADR, of moderate severity. All other AEs related to clinical laboratory test observed in follow-up test conducted 5 days after single administration. They were mild in severity and were judged not relevant with dotinurad by principal investigators.

**Table 4:** Summary of adverse events

Adverse events	Renal function		
	Normal	Mild dysfunction	Moderate dysfunction
	(*n* = 6)	(*n* = 6)	(*n* = 6)
All	0 (0.0%)	0 (0.0%)	2 (33.3%)
Musculoskeletal and connective tissue disorders			
Gouty arthritis	0 (0.0%)	0 (0.0%)	1 (16.7%)
Investigations			
Aspartate aminotransferase increase	0 (0.0%)	0 (0.0%)	1 (16.7%)
Beta 2 microglobulin urine increase	0 (0.0%)	0 (0.0%)	1 (16.7%)
Beta-*N*-acetyl-d-glucosaminidase increase	0 (0.0%)	0 (0.0%)	1 (16.7%)
Blood creatine phosphokinase increase	0 (0.0%)	0 (0.0%)	1 (16.7%)
Gamma-glutamyltransferase increase	0 (0.0%)	0 (0.0%)	1 (16.7%)

## Discussion

This was the first pharmacological study to investigate the PK, PD, and safety of dotinurad in subjects with renal dysfunction.

Regarding PK, we observed that dotinurad concentration profiles and PK parameters of dotinurad and its major metabolites in the mild and moderate renal dysfunction groups were not significantly different from those in the normal renal function group. These findings suggest that the PK of dotinurad is not affected by mild or moderate renal dysfunction. Regarding PD, dotinurad increased urinary uric acid excretion and concomitantly reduced serum uric acid levels, even in subjects with moderate renal dysfunction. ΔEC_max_ and ΔAUEC_0-48_ in the renal dysfunction groups were not significantly different from those in the normal renal function group. Conversely, some PD parameters (the maximum reduction rate in serum uric acid levels and FE_0–24_/FE_−24–0_) were significantly lower in the moderate renal dysfunction group than in the normal renal function group. Regarding safety, gouty arthritis was observed as an ADR in the moderate renal dysfunction group alone (one event in one subject). Otherwise, no safety issues were observed related to clinical examination, vital signs, and ECGs. In addition, in a subgroup analysis based on renal function (normal function and mild or moderate dysfunction) in phase II and phase III studies of patients with hyperuricemia with or without gout, the serum uric acid-lowering effect and safety of dotinurad were barely affected by mild or moderate renal dysfunction [NCT 02416167, NCT 03006445, NCT 03100318, NCT 03372200]. From these results, it is undeniable that dotinurad may be affected by moderate renal dysfunction, but this seemed to have no significant effect. Therefore, dose adjustment may not be necessary when administering dotinurad to patients with mild-to-moderate renal dysfunction.

## Electronic supplementary material

Below is the link to the electronic supplementary material.

Supplementary file1 (DOCX 41 kb)

## References

[1] Loeb JN (1972). The influence of temperature on the solubility of monosodium urate. Arthritis Rheum..

[2] Mikkelsen WN, Valkenburg H (1965). The distribution of serum uric acid values in a population unselected as to gout or hyperuricemia. Am J Med..

[3] Weiner DE, Tighiouart H, Elsayed EF (2008). Uric acid and incident kidney disease in the community. J Am Soc Nephrol..

[4] Tomita M, Mizuno S, Murayama T (2000). Dose hyperuricemia affect mortality? A prospective cohort study of Japanese male workers. I Epidemiol..

[5] Boss GR, Seegmiller JE (1979). Hyperuricemia and gout; classification, complications and management. N Engl J Med..

[6] Mikuls TR, MacLean CH, Olivieri J (2004). Quality of care indicators for gout management. Arthritis Rheum..

[7] Japanese Society of Gout and Uric & Nucleic Acids: guideline for the management of hyperuricemia and gout: 2nd edition. 2010. 10.1080/15257770.2011.596496 22132951

[8] Gillen M, Valdez S, Shen Z (2016). Effects of renal function on pharmacokinetics and pharmacodynamics of lesinurad in adult volunteers. Drug Des Devel Ther..

[9] Lee MH, Graham GG, Day RO (2008). A benefit-risk assessment of benzbromarone in the treatment of gout. Was its withdrawal from the market in the best interest of patients?. Drug Saf..

[10] Chonchol M, Shlipak MG, Fried LF (2007). Relationship of uric acid with progression of kidney disease. Am J Kidney Dis..

