# Supplementary material for: Effects of mild and moderate renal dysfunction on pharmacokinetics, pharmacodynamics, and safety of dotinurad: a novel selective urate reabsorption inhibitor
Source: Clin Exp Nephrol. 2019 Dec 10;24(Suppl 1):17–24. doi: 10.1007/s10157-019-01825-3 (PMC7066277; doi:10.1007/s10157-019-01825-3)
Supplement: Supplementary file 1 — Supplementary file1 (DOCX 41 kb) [file 10157_2019_1825_MOESM1_ESM.docx]

Supplement 1 List of demographic data

| Renal function | Subject ID | Sex | Age*  (year) | Height  (cm) | Weight  (kg) | BMI  (kg/m^2^) | eGFR  (mL/min/1.73 m^2^) |
| --- | --- | --- | --- | --- | --- | --- | --- |
|  | | | | | | | |
| Normal | X1 | Male | 22 | 174.4 | 71.7 | 23.5 | 100 |
|  | X2 | Male | 22 | 164.2 | 64.3 | 23.8 | 111 |
|  | X3 | Male | 22 | 162.9 | 53.3 | 20.0 | 101 |
|  | X4 | Male | 26 | 177.5 | 67.6 | 21.4 | 109 |
|  | X5 | Male | 22 | 169.7 | 59.1 | 20.5 | 99 |
|  | X6 | Male | 22 | 174.7 | 64.2 | 21.0 | 93 |
|  | | | | | | | |
| Mild dysfunction | Y1 | Male | 48 | 163.0 | 63.8 | 24.0 | 61 |
|  | Y2 | Male | 67 | 166.0 | 57.9 | 21.0 | 65 |
|  | Y3 | Male | 30 | 169.3 | 71.9 | 25.0 | 74 |
|  | Y4 | Male | 58 | 152.3 | 59.0 | 25.4 | 69 |
|  | Y5 | Male | 30 | 185.9 | 85.9 | 24.8 | 66 |
|  | Y6 | Male | 63 | 166.0 | 56.1 | 20.3 | 64 |
|  | | | | | | | |
| Moderate dysfunction | Z1 | Male | 75 | 159.5 | 61.3 | 24.0 | 48 |
|  | Z2 | Male | 74 | 169.9 | 62.6 | 21.6 | 58 |
|  | Z3 | Male | 70 | 165.6 | 65.3 | 23.8 | 30 |
|  | Z4 | Male | 79 | 163.7 | 56.6 | 21.1 | 57 |
|  | Z5 | Male | 76 | 173.2 | 59.5 | 19.8 | 58 |
|  | Z6 | Male | 50 | 164.8 | 66.1 | 24.3 | 55 |
| *, At informed consent. | | | | | | | |

Supplement 2 List of the plasma and urine PK parameters of dotinurad

| Renal function | Subject ID | Plasma | | | | | | | | Urine | |
| --- | --- | --- | --- | --- | --- | --- | --- | --- | --- | --- | --- |
|  |  | C_max_  (ng/mL) | T_max_  (hr) | T_1/2_  (hr) | AUC_0-inf_  (ng·hr/mL) | CL_tot_/F  (L/hr) | kel  (1/hr) | Vd/F  (L) | MRT_0-t_  (hr) | Ae_0-24_  (μg) | fe_0-24_  (%) |
|  |  |  |  |  |  |  |  |  |  |  |  |
| Normal | X1 | 82.9 | 3.0 | 10.9 | 1194.8 | 0.84 | 0.064 | 13.1 | 13.1 | 6.8 | 0.678 |
|  | X2 | 87.3 | 3.0 | 8.4 | 1115.3 | 0.90 | 0.083 | 10.9 | 11.6 | 9.2 | 0.916 |
|  | X3 | 98.3 | 4.0 | 8.3 | 1242.9 | 0.80 | 0.084 | 9.6 | 11.7 | 10.8 | 1.081 |
|  | X4 | 66.5 | 4.0 | 6.3 | 721.9 | 1.39 | 0.110 | 12.5 | 10.1 | 7.9 | 0.788 |
|  | X5 | 89.0 | 3.0 | 10.9 | 1558.3 | 0.64 | 0.064 | 10.0 | 13.9 | 7.2 | 0.724 |
|  | X6 | 90.0 | 4.0 | 7.8 | 1110.7 | 0.90 | 0.088 | 10.2 | 12.0 | 16.1 | 1.609 |
|  |  |  |  |  |  |  |  |  |  |  |  |
| Mild dysfunction | Y1 | 79.8 | 3.0 | 10.0 | 1300.0 | 0.77 | 0.069 | 11.1 | 13.0 | 7.1 | 0.712 |
|  | Y2 | 114.0 | 2.0 | 12.2 | 1965.1 | 0.51 | 0.057 | 9.0 | 13.9 | 11.0 | 1.101 |
|  | Y3 | 88.8 | 1.0 | 9.8 | 1169.0 | 0.86 | 0.071 | 12.0 | 11.7 | 14.0 | 1.401 |
|  | Y4 | 105.5 | 3.0 | 10.8 | 1517.3 | 0.66 | 0.064 | 10.2 | 13.8 | 4.9 | 0.487 |
|  | Y5 | 49.4 | 6.0 | 7.8 | 691.8 | 1.45 | 0.089 | 16.3 | 11.2 | 7.6 | 0.758 |
|  | Y6 | 94.9 | 3.0 | 11.2 | 1556.3 | 0.64 | 0.062 | 10.4 | 14.6 | 8.2 | 0.821 |
|  |  |  |  |  |  |  |  |  |  |  |  |
| Moderate dysfunction | Z1 | 81.6 | 3.0 | 12.6 | 1519.3 | 0.66 | 0.055 | 12.0 | 14.4 | 3.8 | 0.379 |
|  | Z2 | 83.4 | 2.0 | 11.0 | 1306.5 | 0.77 | 0.063 | 12.2 | 12.9 | 5.4 | 0.544 |
|  | Z3 | 96.8 | 3.0 | 12.5 | 1858.4 | 0.54 | 0.055 | 9.7 | 14.5 | 5.1 | 0.510 |
|  | Z4 | 108.5 | 2.0 | 11.4 | 1609.4 | 0.62 | 0.061 | 10.2 | 13.2 | 4.0 | 0.397 |
|  | Z5 | 71.6 | 3.0 | 7.3 | 849.1 | 1.18 | 0.095 | 12.3 | 9.5 | 7.9 | 0.786 |
|  | Z6 | 89.5# | 2.0# | 12.3# | 1505.7# | 0.66# | 0.056# | 11.8# | 14.0# | 11.6# | 1.158# |
| #, Non-aggregated data  Ae_0-24_, amount of dotinurad excreted in urine 0 to 24 hour after administration. | | | | | | | | | | | |

Supplement 3 List of the plasma and urine PD parameters of dotinurad

| Renal function | Subject ID | Plasma | | | Urine | | |
| --- | --- | --- | --- | --- | --- | --- | --- |
|  |  | ΔAUEC_0-48_  (mg·hr/dL) | ΔEC_max_  (mg/dL) | Maximum reduction rate  (%) | Ae_0-24, ua_  (mg) | CL_R0-24_  (mL/min) | FE_0-24_  (%) |
|  |  |  |  |  |  |  |  |
| Normal | X1 | −55.4 | −1.5 | 23.4 | 987.9 | 12.9 | 10.1 |
|  | X2 | −71.9 | −2.2 | 33.8 | 1030.3 | 15 | 12.8 |
|  | X3 | −78.5 | −2.2 | 40.0 | 838.2 | 15.2 | 13 |
|  | X4 | −62.0 | −1.8 | 31.6 | 862.8 | 14 | 11.2 |
|  | X5 | −89.7 | −2.4 | 43.6 | 961.3 | 18.7 | 17.2 |
|  | X6 | −83.1 | −2.3 | 39.0 | 1203.5 | 20.4 | 15.6 |
|  |  |  |  |  |  |  |  |
| Mild dysfunction | Y1 | −73.4 | −1.8 | 27.7 | 1044.5 | 14.7 | 13.5 |
|  | Y2 | −116.7 | −2.8 | 32.2 | 1084.8 | 11.6 | 11.4 |
|  | Y3 | −86.4 | −2.1 | 30.0 | 1027.2 | 13.8 | 11 |
|  | Y4 | −95.6 | −2.5 | 37.9 | 909.5 | 13.5 | 13.7 |
|  | Y5 | −37.3 | −1.3 | 31.7 | 816.5 | 17.3 | 19.3 |
|  | Y6 | −65.4 | −1.9 | 44.2 | 1014.5 | 23.9 | 27.8 |
|  |  |  |  |  |  |  |  |
| Moderate dysfunction | Z1 | −56.8 | −1.6 | 34.0 | 795.5 | 15.1 | 23.2 |
|  | Z2 | −36.2 | −1.1 | 14.7 | 829.9 | 8.7 | 9.2 |
|  | Z3 | −41.2 | −1.2 | 11.0 | 703.7 | 4.8 | 10 |
|  | Z4 | −71.7 | −2.1 | 30.9 | 930 | 12 | 15.4 |
|  | Z5 | −41.8 | −1.1 | 15.9 | 799.6 | 9.2 | 13 |
|  | Z6 | 60.2# | −0.2# | 5.7# | 557.7# | 9.9# | 9.6# |
| #, Non-aggregated data  Ae_0-24, ua_, amount of uric acid excreted in urine 0 to 24 hour after administration. | | | | | | | |

Supplement 4 List of adverse events

| Renal function  Subject ID | Adverse events | Date of administration | Date of onset | Date of outcome | Seriousness | Severity | Outcome | Causal relationship |
| --- | --- | --- | --- | --- | --- | --- | --- | --- |
| Moderate dysfunction |  |  |  |  |  |  |  |  |
| Z3 | Gouty arthritis | 2015/01/30 | 2015/02/01 | 2015/02/18 | Non-serious | Moderate | Disappeared | Unlikely related |
|  | Aspartate aminotransferase increased | 2015/01/30 | 2015/02/04 | 2015/02/09 | Non-serious | Mild | Disappeared | No related |
|  | Beta-N-acetyl-D-glucosaminidase increased | 2015/01/30 | 2015/02/04 | 2015/02/09 | Non-serious | Mild | Disappeared | No related |
|  | Gamma-glutamyltransferase increased | 2015/01/30 | 2015/02/04 | 2015/02/20 | Non-serious | Mild | Resolving | No related |
|  |  |  |  |  |  |  |  |  |
| Z5 | Blood creatine phosphokinase increased | 2015/01/30 | 2015/02/04 | 2015/02/11 | Non-serious | Mild | Disappeared | No related |
|  | Beta 2 microglobulin urine increased | 2015/01/30 | 2015/02/04 | 2015/02/11 | Non-serious | Mild | Disappeared | No related |
